# An Intelligent Auxiliary Framework for Bone Malignant Tumor Lesion Segmentation in Medical Image Analysis

**DOI:** 10.3390/diagnostics13020223

**Published:** 2023-01-07

**Authors:** Xiangbing Zhan, Jun Liu, Huiyun Long, Jun Zhu, Haoyu Tang, Fangfang Gou, Jia Wu

**Affiliations:** 1 State Key Laboratory of Public Big Data, College of Computer Science and Technology, Guizhou University, Guiyang 550025, China; 2 The Second People’s Hospital of Huaihua, Huaihua 418000, China; 3 The First People’s Hospital of Huaihua, Huaihua 418000, China; 4 Collaborative Innovation Center for Medical Artificial Intelligence and Big Data Decision Making Assistance, Hunan University of Medicine, Huaihua 418000, China; 5 Research Center for Artificial Intelligence, Monash University, Melbourne, VIC 3800, Australia

**Keywords:** bone malignant tumor lesion, supervised edge attention, boundary key points, medical image, intelligent auxiliary

## Abstract

Bone malignant tumors are metastatic and aggressive, with poor treatment outcomes and prognosis. Rapid and accurate diagnosis is crucial for limb salvage and increasing the survival rate. There is a lack of research on deep learning to segment bone malignant tumor lesions in medical images with complex backgrounds and blurred boundaries. Therefore, we propose a new intelligent auxiliary framework for the medical image segmentation of bone malignant tumor lesions, which consists of a supervised edge-attention guidance segmentation network (SEAGNET). We design a boundary key points selection module to supervise the learning of edge attention in the model to retain fine-grained edge feature information. We precisely locate malignant tumors by instance segmentation networks while extracting feature maps of tumor lesions in medical images. The rich contextual-dependent information in the feature map is captured by mixed attention to better understand the uncertainty and ambiguity of the boundary, and edge attention learning is used to guide the segmentation network to focus on the fuzzy boundary of the tumor region. We implement extensive experiments on real-world medical data to validate our model. It validates the superiority of our method over the latest segmentation methods, achieving the best performance in terms of the Dice similarity coefficient (0.967), precision (0.968), and accuracy (0.996). The results prove the important contribution of the framework in assisting doctors to improve the accuracy of diagnosis and clinical efficiency.

## 1. Introduction

Bone malignant tumors have always been one of the most serious problems of human health [1]. They can be divided into primary and metastatic bone tumors. The disease is life-threatening and difficult to treat. The most frequent bone malignant tumor is osteosarcoma, and the second is Ewing’s sarcoma [2]. The two types of sarcomas described above are primary bone malignant tumors that are more prevalent in adolescents and have high metastatic rates and mortality rates [3]. If patients are not diagnosed and treated in a timely manner, they may be at risk of amputation or metastasis. Therefore, early detection and timely treatment of the disease will increase the survival rate.

When a physician lacks sufficient experience, it is difficult to detect and segment bone malignant lesions accurately in real time using traditional diagnostic methods based on manual labor. Only about 20 of the approximately 600 MRI images of a patient contain lesions. Inexperienced doctors may take 10 s to review each medical image and 3–5 min to review all the images. Clinical efficiency is extremely low when there are a lot of patients. Since 10% of bone tumors are malignant and there have been fewer cases in the past, detection and segmentation require doctors with sophisticated expertise as well as substantial experience [4]. The diagnostic process can be time-consuming and laborious, making it difficult to improve the accuracy and efficiency of diagnosis. Furthermore, there are limitations on healthcare resources and poor construction of medical infrastructure in low-income areas of developing countries, as well as an inequitable distribution of public healthcare resources between developed cities and remote areas. In China, for instance, about 80% of medical resources are centered in developed cities, where only 10% of the population is located. However, 90% of those living in remote mountainous areas receive only 20% of healthcare resources [5,6,7,8]. Therefore, there is an urgent need for practical solutions to address these issues. This is why we propose an intelligent auxiliary framework for segmenting bone malignant tumors.

AI has a wide range of applications in the healthcare industry and can significantly enhance clinical diagnosis efficiency by compensating for the shortage of healthcare resources and doctors’ experience. Currently, deep learning is being extensively applied to medical image analysis in the field of diseases [9,10,11] and tumors, such as lung cancer [12,13] and breast cancer [5,14,15]. Medical AI systems based on deep learning have similarly shown significant promise in aiding the diagnosis and prognosis prediction of bone tumors based on imaging scans such as X-rays, CT, MRI, PET, and histopathology slides [16]. As presented in [17], the authors propose a multi-task deep learning model for segmenting and classifying primary bone tumors on radiographs with a performance equivalent to that of a trained doctor. To assist doctors, the authors in [18] used deep learning to classify and segment knee bone tumors in X-ray images. It should be noted, however, that these studies were limited to X-ray images without considering MRI data.

In clinical practice, the accurate localization of tumor lesion areas and boundaries from medical images plays a crucial role in diagnosing disease, making medical decisions, and developing treatment plans. In particular, MRI is non-radioactive and non-biologically damaging to the body. It is more effective for soft tissue components such as tumors, blood vessels, and muscles. Therefore, MRI is an effective diagnostic and evaluation tool for doctors. According to our knowledge, no relevant studies have been conducted in the area of segmenting the most common malignant bone tumors in MRI. This paper presents for the first time an intelligent auxiliary framework that fills an existing gap in the field.

Deep learning is being applied to the field of medical image segmentation in order to assist doctors in identifying disease lesions efficiently and accurately [19,20,21,22]. There is also the network architecture U-Net [23] for biomedical image segmentation, and its variants UNet++ [24], UNet 3+ [25]. These methods are suitable only for segmentation tasks in which anatomical structures are clearly defined. Among the primary bone malignant tumors, osteosarcoma is the most common. The tumor lesion boundaries on MRI images are ambiguous and the background relationships are complex. Therefore, we propose the use of a supervised approach to achieve accurate segmentation of the lesions. Osteosarcoma has a low survival rate [26] and is prone to metastasis to the lungs [27,28,29,30], and if diagnosed in time, the 5-year survival rate rises to 74% [31,32]. Based on MRI images of osteosarcoma, we propose an intelligent auxiliary framework for segmenting bone malignant lesions, aiming to improve the accuracy and efficiency of clinical diagnosis for doctors.

The main contributions of this study are as follows:

1. We propose SEAGNET, a novel segmentation network, for the accurate segmentation of bone malignant tumor lesions in medical images.
2. We develop a new edge attention module that enables the network to focus on the fuzzy boundaries of real tumor lesions, which captures more edge detail feature information.
3. We design a boundary key points selection algorithm to encode the selected key points on the tumor boundary, which supervises the learning of edge attention to correct the segmentation of the network.
4. We validate the proposed model with a wide range of experiments on a real-world dataset. The results show that it outperforms other models in the segmentation of bone malignant tumor lesions with complex backgrounds and blurred boundaries.

## 2. Related Work

Although there are some methods which focus on the boundary [33,34] or the edge [35,36], these methods are based on the segmentation of medical images of skin lesions, blood vessels, and lungs. It is difficult to apply these methods to the segmentation of targets with blurred boundaries and complex background relationships in medical images.

In the study of bone tumors based on X-ray images, Furuo et al. [4] used deep learning to automatically classify tumors as benign or malignant: VGG16 (f1 score: 0.790) and ResNet152 (f1 score: 0.784). von Schacky et al. [17] proposed a multi-task deep learning model with 80.2% accuracy, 62.9% sensitivity, and 88.2% specificity in the classification of tumors as benign or malignant using bounding-box placement IoU (0.52) and the mean Dice score (0.6) for segmentation. In [18], the authors proposed the Seg-Unet model for the segmentation of knee bone tumors (mean IoU of 84.84%) as well as for classifying the tumors as benign or malignant (99.05% accuracy). Since the tissue structures in single-view X-ray images are overlapping and have limited resolution, unlike MRI images which have excellent resolution and rich texture details of the tissue, it is difficult for the model to capture recognizable features, which limits its performance.

Dionisio et al. [3] demonstrated a high similarity between manual and semi-automatic segmentation of bone sarcomas in MRI. However, their semi-automatic segmentation method is based on the GrowCut tool, which requires the help of manual labor, rather than a deep learning model for automatic segmentation. Currently, machine learning is being applied to primary bone malignant tumors, such as osteosarcoma.

The following studies considered the detection and classification of osteosarcomas based on histological images. Based on the transformer deep learning technique, Pan et al. [37] classified necrotic tumors, non-tumors, and viable tumors (99.17% accuracy). However, the model size is large, and training requires advanced hardware equipment and a lot of time. Again, Badashah et al. [38] proposed a GAN based on fractional-Harris hawks optimization, achieving 98% accuracy, sensitivity, and specificity, but the dataset is small, and the model is difficult to scale up to large datasets. In addition, Anisuzzaman et al. [39] adopted pre-trained CNNs (VGG19 and Inception V3) and transfer learning techniques. They achieved the highest accuracy (96%) with VGG19 but the results were not evaluated by pathologists. A combination of traditional manual features and deep learning features was used to achieve an overall accuracy of 99.54% by Bansal et al. [32]. All of these studies identify the three types of osteosarcoma in histological images that require needle biopsy. There have also been some exploratory studies in the management of treatment response in osteosarcoma [40,41,42,43]. However, this requires specialized biomedical knowledge.

For segmentation, Baidya Kayal et al. [44] used DWI data. The DNN has the highest accuracy (Dice coefficient: ~73%; Jaccard index: ~62%; precision: ~77%; recall: ~86%; Pearson correlation coefficient = 0.79). However, the model is very sensitive to the noise in the image as well as the initialization of the algorithm. Nasor et al. [45] integrated common image processing techniques such as Canny edge detection, Gaussian filtering, and machine learning techniques such as the K-means clustering algorithm, resulting in 95.96% precision, 86.15% recall, 99.51% specificity, an 89.84% Dice score coefficient, and 98.02% accuracy, but the test data is only 50 images, and the model is only applicable to simple tasks.

In addition, Wu et al. [46] considered the relationship between body features and edge features. With fewer parameters, the percentage of IOU is 90.51%. It can be challenging to process redundant information when both edge features and full-image information are taken into consideration. In another work [47], they proposed that the Residual Fusion Network learns image information at different resolutions (DSC: 0.929; IOU: 0.867). Understanding the consistency of information at different resolutions can lead to more complex models that are prone to overfitting. Liu et al. [48] proposed the CaPaN to efficiently encode tumor lesions of different sizes and feature local to global information. There is a greater advantage (DSC of 0.913) in segmenting small target tumors. However, the domain adaptivity between multi-scale targets is not sufficiently considered. Wu et al. [49] proposed the combination of SepUNet and CRF for the segmentation of tumor regions. Although speed and cost are taken into account to ensure accuracy (DSC: 0.914), the excessive pre-processing of raw data not only leads to a loss of information but also reduces the error tolerance of the model for sensitive data. Shen et al. [50] introduced the FaBiNet to learn local detail features and global top information separately, finally integrating them for the segmentation task with 0.95 accuracy and 2.33 M params. Nevertheless, the lack of an inherently consistent understanding of high-level semantic information and low-level information causes performance limitations.

Moreover, the effective use of advanced transformer technology and a self-attention mechanism, together with noise pre-processing techniques, brings great benefits to medical image segmentation. Wu et al. [51] proposed ETUNet for the segmentation of osteosarcoma lesions after filtering medical images with redundancy and noise to improve the segmentation performance (DSC of 0.935). However, the use of subjective assessments in pre-processing and noise reduction is subject to bias and error. Wang et al. [52] took into account the location and regional information of the tumor from the perspective of local enhancement after processing the noise; the proposed DFANet achieves excellent segmentation performance (DSC of 0.964). Introducing complex network modules in noise reduction can lead to bloated models with too large scales. To capture medical image features globally, Ling et al. [53] used CNN and Swin Transformer. The results show that DSC outperforms Unet by 2.6% and Unet++ by 1.8%. However, Swin Transformer will generate huge memory consumption for large-scale resolutions. Ouyang et al. [54] designed a segmentation architecture (UATransNet) that fuses local to global features based on self-attention and U-Net to improve the accuracy of segmentation (IOU: 0.922 ± 0.03, DSC: 0.921 ± 0.04). This method cannot accurately segment fuzzy boundaries with little difference in gray level. Lv et al. [55] proposed a priori guided segmentation networks based on few-shot learning with a DSC of 0.945. However, few-shot learning tends to be limited to domain-specific problems. Wu et al. [56] presented BA-GCA Net to learn better texture detail information of tumor region boundaries in order to achieve better performance, with a DSC of 0.927. In the case of simple tasks, however, the model trained for complex scenarios will easily overfit. The transformer technique improves the performance of segmentation, but overly complex deep learning models are more difficult to train and tune, as well as prone to overfitting.

Unlike the above methods, we focus more on understanding the uncertainty and ambiguity of fuzzy boundaries in medical images with complex backgrounds, and the inherent consistency of image features in order to better segment the boundaries of tumors. We develop an intelligent auxiliary framework for segmenting bone malignant tumors. The purpose of this is to compensate for the lack of clinical experience of doctors and to optimize the diagnostic workflow. Since osteosarcoma is very typical and most frequent among bone malignant tumors, it seriously endangers the life and health of children as well as adolescents. Therefore, we adopted osteosarcoma in MRI images as the case study for this paper.

## 3. Framework Design

**Figure 1:**
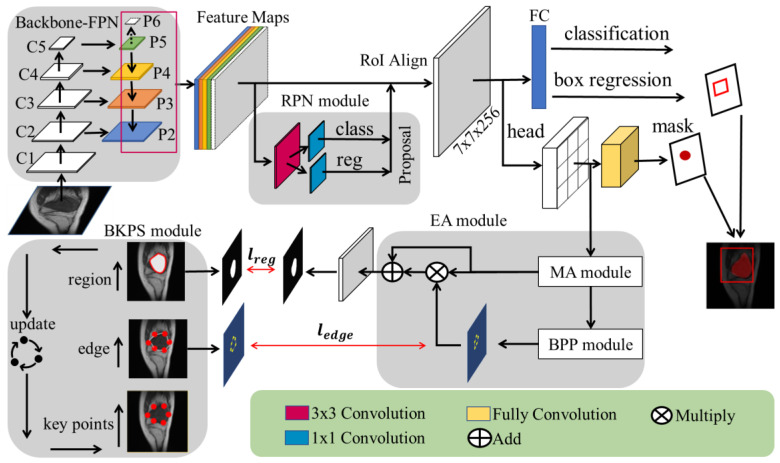
The architecture diagram of SEAGNET.

This section introduces the framework of the SEAGNET for the precise segmentation of bone tumors in MRI. Firstly, the feature maps containing low-level features and high-level semantic information were obtained from the output {P2, P3, P4, P5, P6} of the Feature Pyramid Network (FPN) with ResNet-50 as the backbone, as shown in Figure 1. Secondly, all candidate boundary boxes of regions of interest (RoIs) are filtered by a Region Proposal Network (RPN [57]) module. The RoI Align operation will faithfully preserve the accuracy of spatial locations; the pixel positions are precisely aligned in space, and then boundary-box regression and mask generation are performed to localize the region of the bone tumor lesion. A mixed attention (MA) module captures abundant context-dependent information about the tumor lesion region and its edge background from the generated mask head. In the edge attention (EA) module, the predicted boundary points column feature maps are generated by the boundary points proposal (BPP) module, and edge attention is learned so as to weight the extracted features to guide segmentation. Finally, a boundary key points selection (BKPS) module is used to supervise the learning of edge attention, and then capture the fine-grained feature information of the ambiguous boundary of the lesion region.

In the overall structure diagram of SEAGNET, firstly, the upper part of the diagram shows the instance-segmentation architecture that locates the position and region of the bone tumor. It also includes the regression of the bounding box and the generation of the head mask. The purpose is to obtain a feature map of the head mask. Secondly, the network branch with the head mask features as input for the segmentation network for bone malignant tumor lesions.

### 3.1. The RoI Align Operation

**Figure 2:**
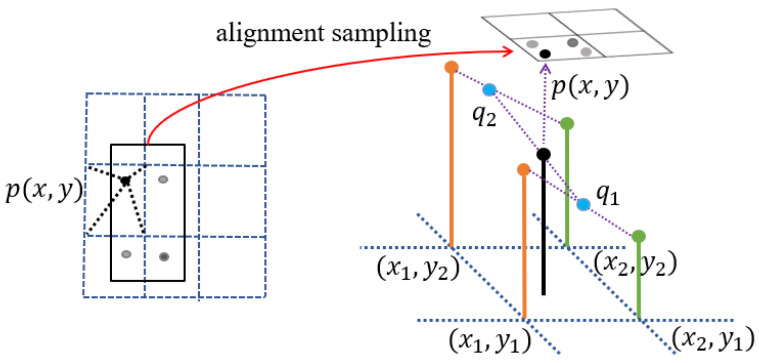
The alignment sampling in the RoI Align operation. A window is set up with four sampling points. Each sampled point’s value is computed by bilinear interpolation from the four adjacent pixel points covered by the window.

The process of extracting the head mask features using the instance segmentation network enables the preservation of important pixel spatial location information on the bone tumor edges. We follow the RoI Align concept in Mask R-CNN [58] and add a layer of alignment sampling for the pixel points that cannot be integrable when obtaining the small feature map (7 × 7) of the proposal bounding box, as shown in Figure 2. Pooling is performed after sampling by bilinear interpolation, instead of simply performing the maximum pooling operation. Maximum pooling ignores the key pixels with strong discriminative power near the fuzzy boundary of bone malignant tumors, which will result in the loss of accurate spatial location information. This is negative for the accurate prediction of the pixel mask which will limit the performance of bone tumor segmentation.

Given that the pixel values of the four nearby grid points are $f\left(x_{1},y_{1}\right)$, $f\left(x_{1},y_{2}\right)$, $f\left(x_{2},y_{1}\right)$, $f\left(x_{2},y_{2}\right)$, then the pixel value of $p\left(x,y\right)$ is:

(1) $$\begin{array}{c} f\left(p\right)=\frac{y_{2}\;-\;y}{y_{2}\;-\;y_{1}}f\left(q_{1}\right)+\frac{y\;-\;y_{1}}{y_{2}\;-\;y_{1}}f\left(q_{2}\right) \end{array}$$

The pixel values of $q_{1}$, $q_{1}$ are:

(2) $$\begin{array}{c} f\left(q_{1}\right)=\frac{x_{2}\;-\;x}{x_{2}\;-\;x_{1}}f\left(x_{1},y_{1}\right)+\frac{x\;-\;x_{1}}{x_{2}\;-\;x_{1}}f\left(x_{2},y_{1}\right) \end{array}$$

(3) $$f\left(q_{2}\right)=\frac{x_{2}\;-\;x}{x_{2}\;-\;x_{1}}f\left(x_{1},y_{2}\right)+\frac{x\;-\;x_{1}}{x_{2}\;-\;x_{1}}f\left(x_{2},y_{2}\right)$$

### 3.2. The Edge Attention Module

Using edge attention, the network is guided to focus on the boundaries of tumor lesions, exploring discriminative pixel information, and avoiding background noise interference. The module first learns the relevant dependency information of the features in the mask head using the mixed attention module to obtain an enhanced feature representation $F_{tumor}\in {\mathbb{R}}^{w\times h\times c}$ for the inherent understanding of the boundary fuzzy features. Then, the boundary points proposal (BPP) module learns the edge key pixel points feature map $F_{points}\in {\mathbb{R}}^{w\times h\times 1}$, which contains the weights of the edge pixels, and the $F_{tumor}$ weights to guide structural boundary information learning. To avoid the impact of attention features on the performance of the original $F_{tumor}$, a skip connection is used to fuse $F_{tumor}$ and $F_{points}$, which generates the edge attention-enhanced feature map $F_{edge}\in {\mathbb{R}}^{w\times h\times c}$. Its mathematical representation is:

(4) $$\begin{array}{c} F_{edge}=F_{tumor}⊗\left(1⊕F_{points}\right) \end{array}$$

where ⊗ denotes multiply and ⊕ denotes add. If the results of the attention $F_{tumor}⊗F_{points}$ are close to 0, then the $F_{edge}$ is approximately equal to the $F_{tumor}$ which preserves the original features, and the real role of the attention is to enhance the features of the fuzzy borders of the bone tumor lesion.

#### 3.2.1. The Mixed Attention Module

**Figure 3:**
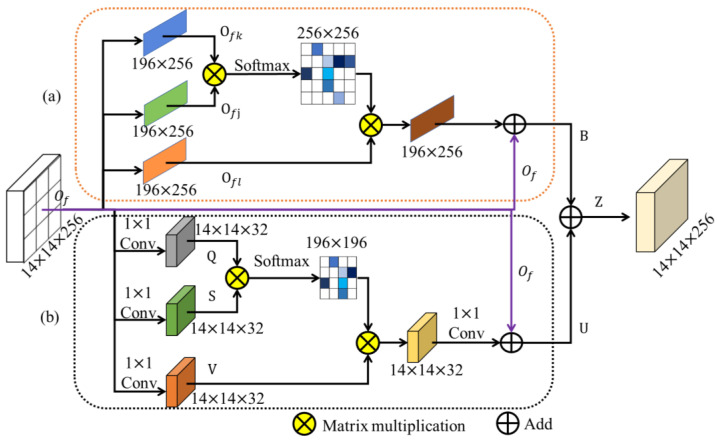
This figure introduces the mixed attention module of the SEAGNET. (**a**) Denotes the channel attention module and (**b**) denotes the spatial attention module.

The feature map of the head mask contains information about bone tumor lesions, nearby tissues, and the background. Additionally, the segmentation task does not only focus on the regions of bone tumor lesions, but also on other structures within the medical image, such as muscles, fat, healthy bone, and other tissues. For this reason, we propose a mixed attention module that captures rich remote context dependencies from both the channel dimension and the spatial dimension to better understand the uncertainty and ambiguity of fuzzy boundaries. As shown in Figure 3, the module architecture consists of two branches: the channel attention branch and the spatial attention branch.

The channel attention branch directly reshapes the head mask features $O_{f}\in {\mathbb{R}}^{H\times W\times C}$ into ${\mathbb{R}}^{N\times C},\;N=H\times W$, preserving the original number of channels. Then the attention weights $A\in {\mathbb{R}}^{C\times C}$ between each channel feature are calculated:

(5) $$\begin{array}{c} a_{jk}= \end{array}exp\left(O_{fk}\;\cdot \;O_{fj}\right)/\sum_{k=1}^{C}exp\left(O_{fk}\;\cdot \;O_{fj}\right)$$

where $a_{jk}$ is the impact of channel k on channel *j*.

The features of the bone tumor are soft weighted $a_{jk}\;\cdot \;O_{fl}$, and other instance features are given very small weights for filtering, thus avoiding the interference of complex background noise information. Finally, the features are fused with the original features by skip connection to enhance the discriminative ability of bone tumor features in the channels, the output is $B\in {\mathbb{R}}^{H\times W\times C}$:

(6) $$\begin{array}{c} B_{j}=\omega \sum_{k=1}^{C}\left(a_{jk}\;\cdot \;O_{fl}\right)+O_{fj} \end{array}$$

where ω is the learning factor.

The spatial attention branch first performs a channel dimensionality reduction of the input feature $O_{f}\in {\mathbb{R}}^{H\times W\times C}$ by 1 × 1 convolution to produce three new feature maps $\left\{Q,\;S,\;V\right\}\in {\mathbb{R}}^{H\times W\times \frac{C}{8}}$. With a structure similar to that of the channel attention branch, the attention weights $E\in {\mathbb{R}}^{N\times N},\;N=H\times W$ of the relative positions of the features are calculated:

(7) $$\begin{array}{c} e_{jk} \end{array}=exp\left(Q_{k}\;\cdot \;S_{j}\right)/\sum_{k=1}^{N}exp\left(Q_{k}\;\cdot \;S_{j}\right)$$

where $e_{jk}$ is the impact of position k on position j.

Before the relative spatial positions of the features are soft-weighted, we reshape V into ${\mathbb{R}}^{N\times \frac{C}{8}}$. After that, upsampling using 1 × 1 convolution maintains the same dimensionality as the head mask feature map. Finally skip connection is used to fuse the original features and attention features in a residual way. The output of the spatial attention branch is $U\in {\mathbb{R}}^{H\times W\times C}$:

(8) $$\begin{array}{c} U_{j}=\varphi \sum_{k=1}^{N}\left(e_{jk}\;\cdot \;V_{k}\right)+O_{fj} \end{array}$$

where φ is the learning factor.

Lastly, the outputs of the two branching networks are integrated into a new feature map Z=B+U to better understand the inherent consistency of the boundary features and thus enhance the representation of the original features.

#### 3.2.2. The Boundary Points Proposal Module

In this paper, the proposed BPP module is used to predict the boundary feature map of the tumor lesion region in medical images. The network activates pixels with discriminative ability on the edges as candidate boundary points, which locate the position of the fuzzy boundary.

**Figure 4:**
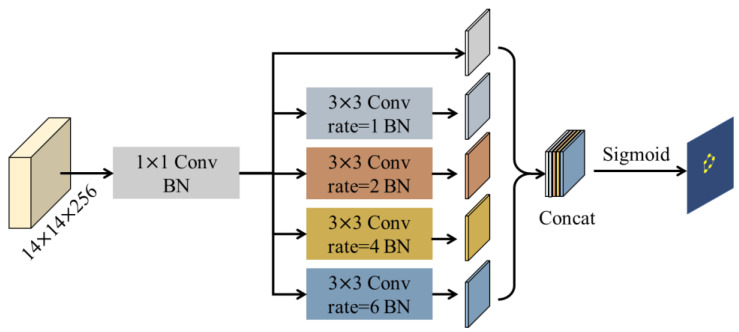
The architecture diagram of the BPP module.

In order to capture the long-range dependence of pixels, we perform dilated convolution to expand the receptive field instead of using pooling, which leads to the loss of precise spatial location information. Moreover, a parallel multi-branch structure is employed to extract features at various resolutions using different scales of dilated convolution. As a result, the network is able to better understand the inherent consistency of fuzzy boundary context features in medical images. This improves the segmentation accuracy of bone malignant tumors. As shown in Figure 4, the module consists of several parallel branches, each of which generates feature maps at different scales. The role of 1 × 1 convolution is to reduce the dimensionality, and the different dilation rate on each branch represents the different scales of dilated convolution (kernel size of 3 × 3) to capture the features under different receptive fields. Each convolution (Conv) operation is appended with batch normalization (BN) for regularization.

Finally, the network concatenates (Concat) the outputs of the different branches, then activates the candidate point maps on the fuzzy boundaries using the Sigmoid function, and outputs the attention matrix of the boundary point columns to weight the original features.

### 3.3. The Boundary Key Points Selection Module

To make the segmentation network converge rapidly and minimize the uncertainty of fuzzy boundaries, the problem of the isolated distribution of fuzzy boundaries of bone malignant tumor lesions in image segmentation is solved. In other words, the edge-attention network alone cannot perceive the complex tumor boundaries in the ambiguous lesion region. Therefore, we use supervised learning to correct the training parameters of the network.

**Figure 5:**
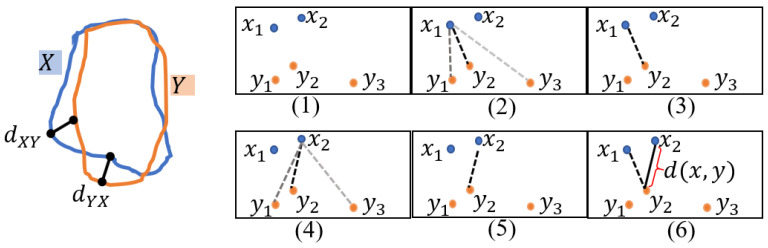
An example of an HD calculation.

Therefore, there is a need to obtain the key points that most closely match the boundaries of real bone tumor lesions in medical images. We designed a boundary key point selection algorithm (Algorithm 1) to solve this problem. The algorithm is trained iteratively to generate the optimal boundary points. First, the boundary of the bone tumor is obtained from the actual MRI image using a traditional edge detection algorithm. Then we randomly select n points $P_{n}^{t}=\left\{\left(x_{1}^{t},y_{1}^{t}\right),\;\left(x_{2}^{t},y_{2}^{t}\right),\cdots ,\;\left(x_{n}^{t},y_{n}^{t}\right)\right\}$, where t represents the number of iterative experiments, and we construct a boundary region $Reg_{n}^{t}$ by connecting these points. We measure the similarity between the constructed boundary region $Reg_{n}^{t}=const\left(P_{n}^{t}\right)$ and the segmentation region $Reg_{GT}$ of the ground truth by computing the Hausdorff distance (HD); the calculation rules for HD are shown in Figure 5. This metric is more sensitive to the boundary of the segmentation region. Finally, the boundary points with the minimum HD value are selected.

The equation for HD can be written as:

(9) $$\begin{array}{c} HD\left(X,Y\right)=\max\left(d_{XY},d_{YX}\right)=\max\left\{\max\min d\left(x,y\right),\max\min d\left(y,x\right)\right\} \end{array}$$

where x∈X denotes the pixel points on the boundary of $Reg_{n}^{t}$ and y∈Y denotes the pixel points on the boundary of $Reg_{GT}$.

The selected key points $P_{select}$ is calculated as:

(10) $$P_{select}=\arg\;\min\;HD\left(Reg_{n}^{t},\;\;\;\;Reg_{GT}\right),\;t\in \left[1,T\right]$$

**Algorithm 1** Boundary Key Points Selection.1: **function** Selection(**T**, $Reg_{GT}$, n)2:   $HD_{min}\leftarrow +\infty$               // Initialization3:   **for**
*t* = 1 to **T do**4:      $P_{n}^{t}\leftarrow \left\{\left(x_{1}^{t},\;y_{1}^{t}\right),\left(x_{2}^{t},\;y_{2}^{t}\right),\cdots ,\left(x_{n}^{t},\;y_{n}^{t}\right)\right\}$  // select n boundary points5:      $Reg_{n}^{t}\leftarrow const\left(P_{n}^{t}\;\right)$           // construct the region6:      $HD_{t}\leftarrow HD\left(Reg_{n}^{t},\;Reg_{GT}\right)$        // Hausdorff Distance7:      **if**  $HD_{t}<HD_{min}$
**then**8:           $HD_{min}\leftarrow HD_{t}$
9:        $P_{select}\leftarrow P_{n}^{t}$              //update10:     **else**11:        continue12:   **return**
$P_{select}$


To eliminate the effect of outliers, the result retains 95% of the distances between the boundary points X and Y. X is the boundary point of the region $Reg_{n}^{t}$, Y is the boundary point of $Reg_{GT}$. On the right side in Figure 5, through steps (1) to (3), we select the minimum distance from point $x_{1}$ in X to all points in Y. Similarly, the minimum distance from the point $x_{2}$ in X to all points in Y is selected from (4) to (5). We convert the roles of X and Y, calculate the bidirectional HD between X and Y, and take the maximum value of the distance to measure the similarity between X and Y. In other words, the smaller the value of HD, the higher the similarity between X and Y. The optimal boundary points are selected by the minimum HD value.

### 3.4. Model Description

The detailed configuration of the model is shown in Table 1, including the main layers, their operations, and their input sizes. First, we obtain the feature maps for performing downstream tasks through the backbone (ResNet50) + FPN architecture: rpn_feature_map and mask_feature_map. Second, we obtain the candidate boxes through the RPN network. RoI Align generates 7 × 7 × 256 feature maps. There are two branches to the network output: one is a shared, fully connected layer that performs classification and box regression respectively, and the other is our head mask feature generation, which is used for downstream tasks in the EA module as input. The final output of the segmentation mask (512 × 512 × 1) and the boundary point attention map (512 × 512 × 1) will be computed with the output of the trained BKPS module for loss. The loss is minimized through training to achieve the purpose of the BKPS module, which is to supervise the EA network and correct the prediction.

### 3.5. Loss Function

We train the instance segmentation task in medical images to capture features accurately localized to the bone tumor lesion region. The loss function is the sum of the losses from multiple tasks:

(11) $$l_{seg}=l_{cls}+l_{box}+l_{mask}$$

(12) $$l_{cls}=-\alpha {\left(1-\rho \right)}^{\gamma }\log\left(\rho \right)$$

where the $l_{cls}$ represents the classification task. The advantage of the loss is to solve the problem of difficult-to-classify samples and improve the performance of the model in general. ρ indicates the affinity of the prediction with ground truth, α and γ are two variable parameters.

The $l_{box}$ is a variant of the IOU loss for tumor lesion detection:

(13) $$\begin{array}{c} l_{box}=l_{IoU}+{\left(\frac{d}{c}\right)}^{p} \end{array}$$

where d denotes the distance between the center point of the prediction box and the ground truth, c is the distance between the diagonal of the smallest convex shapes, and p is the control parameter, $l_{IoU}=1-IOU$.

To decouple from the classification task and highlight the exact contribution of each pixel, a sigmoid activation is used at each pixel and the average binary cross-entropy loss is employed as the mask loss $l_{mask}$.

In the supervised learning of edge attention networks by the BKPS module, we introduce the minimum cross-entropy loss $l_{edge}$ between the distribution $D_{pred}$ predicted by the BPP module and the distribution $D_{GT}$ generated using the key points selection algorithm on the ground truth.

(14) $$l_{edge}=-D_{GT}\cdot \log D_{pred}-\left(1-D_{GT}\right)\cdot \log\left(1-D_{pred}\right)$$

$Reg_{GT}$ constructed from the ground truth and the region $Reg_{pred}$ segmented by the edge-attention guidance is:

(15) $$l_{reg}=-Reg_{GT}\cdot \log Reg_{pred}-\left(1-Reg_{GT}\right)\;\cdot \;\log(1-Reg_{pred})$$

Finally, the total loss of the entire malignant tumor lesions segmentation framework is:

(16) $$l_{total}=l_{seg}+l_{edge}+l_{reg}$$

## 4. Experiments

### 4.1. Datasets

The medical image dataset of bone malignant tumors used in our experiments is over 4000 MRI images of osteosarcoma from 89 patients provided by our domestic partner institution (The Second People’s Hospital of Huaihua), and the ground truth was annotated by three doctors with more than 10 years of clinical experience in hospitals. The annotation of the dataset was conducted in two phases. In the first fully blinded phase, each doctor independently reviewed each osteosarcoma MRI image without interference from other doctors and marked the segmentation mask of the tumor lesion. The second phase involved open review. Each doctor independently reviewed his own segmentation markers as well as the anonymous segmentation markers of the other two doctors and provided his comments. For the annotated data, we selected the MRI images annotated by three physicians in agreement. This was done to avoid potential bias and inconsistency due to the different experiences of the doctors. The ratio of data used for training and testing in the experiment is 8:2. These data include T1 and T2 images from MRI, and the tumor lesion areas are mainly located in the patient’s legs and knees. All data was obtained from within the last 10 years of the hospital’s records.

### 4.2. Evaluation Metrics

In our experiments, we chose several metrics: accuracy (Acc), precision (Pre), recall (Re), F1 score (F1), intersection over union (IOU) and Dice similarity coefficient (DSC), to analyze the performance of the SEAGNET model. In the confusion matrix of the model the metrics were: true positive (*TP*), true negative (*TN*), false positive (*FP*), and false negative (*FN*), where *TP* represents the predicted lesion region, which is the tumor lesions. *TN* represents the indicated normal area, but the actual situation is healthy tissue. *FP* means that the predicted pixels are labeled as a tumor lesion, but they are located in the non-lesion area. *FN* indicates that the predicted pixels are located in the region of healthy tissue, while in fact they are in the tumor region. The equations to calculate Acc, Pre, Re, and F1 are as follows:

(17) $$\mathrm{Acc}=\frac{TP+TN}{TP+TN+FP+FN}$$

(18) $$\mathrm{Pre}=\frac{TP}{TP+FP}$$

(19) Re=TPTP+FN

(20) $$F1=\frac{2*Pre*Re}{Pre+Re}$$

Both IOU and DSC are metrics that measure the similarity between two sets. The range of the values of these two metrics is from 0 to 1, and a value closer to 1 means a segmentation result with close to ground truth. We set $I_{1}$ to represent the predicted pixels belonging to the tumor lesion region, and $I_{2}$ as the ground truth. Then the IOU and DSC are calculated as follows.

(21) $$\mathrm{IOU}=\frac{I_{1}\cap I_{2}}{I_{1}\cup I_{2}}$$

(22) $$\mathrm{DSC}=\frac{2*\left|I_{1}\cap I_{2}\right|}{\left|I_{1}\right|+\left|I_{2}\right|}$$

### 4.3. Training Strategy

The model was optimized using the Adam optimizer and implemented using PyTorch. Our model was trained with 182 epochs. The size of the batch was 16, the momentum and weight decay coefficients were set to 0.9 and 0.0001, respectively, and the initial learning rate was 0.02. The size of the input images was 512 × 512, and we augmented the medical image dataset of tumors by scaling, cropping, rotating, transforming, flipping, and salting.

**Figure 6:**
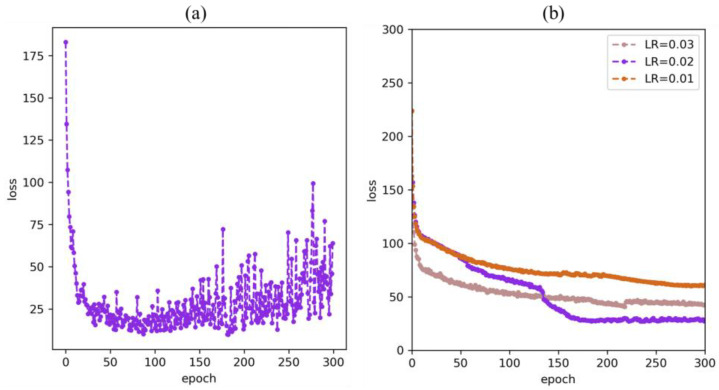
The fine-tuning experiment of model hyperparameters.

To improve the performance of the model we conducted fine-tuning experiments on the hyperparameters. According to Figure 6a, when the model was trained to 182 epochs, the loss was minimized. It was at this point that the model reached its optimal fit. But as the epoch increases, the loss of the model shakes around the optimal point. Therefore, we selected the minimum number of epochs that would achieve the optimal solution. At the same time, we conducted comparison experiments for three learning rates {0.01, 0.02, 0.03} to identify the most appropriate learning rate (LR). The choice of the initial learning rate is crucial to the training of the model. For our task, we performed experiment analysis at the three selectable learning rates mentioned above. The results are shown in Figure 6b. The learning rate (0.01) that is too small has a slow loss drop and continues training does not achieve better results. Although the larger learning rate (0.03) has a fast loss drop, it does not achieve an optimal fit. As for LR=0.02, the loss drop is not obvious at first, but there is a noticeable drop at epoch=50, and the loss drop is more significant at epoch=130 or so until the best-fit point. When the model was trained to 182 epochs, the loss of LR = 0.02 was minimized. As a result, the initial learning rate of our model was determined to be 0.02.

### 4.4. Comparison with Different Methods

To demonstrate the effectiveness of our proposed method, we fine-tuned the model during training. Table 2 shows the superiority of the BKPS module of this method for supervised edge attention learning, which leads to the precise localization and segmentation of tumor fuzzy boundaries. In addition, in the edge attention module, the mixed attention enables the network to better understand the uncertainty and ambiguity of tumor boundaries and to be able to model the rich dependencies of boundary contexts, thus enhancing feature representation and improving segmentation performance. Our method achieves the best performance in three metrics: DSC (0.967), Pre (0.968), and Acc (0.996). The results for IOU, Re, and F1 are equivalent to the best existing segmentation methods for osteosarcoma. Although Eformer + DFANet achieved the best results for IOU (0.928), Re (0.955), and F1 (0.961), our method is close enough to be in second place among all existing methods. Some methods are not given for some metrics, but DSC, as the most important evaluation metric, can be combined with other metrics to evaluate the model comprehensively. This demonstrates that SEAGNET is effective and feasible in identifying the fuzzy boundaries of bone tumor lesions in medical images with complex background correlation.

We also compare the following classical segmentation methods: FCN [59], DeepLab V3+ [60], and U-Net [23]. More information is listed below.

(1) FCN takes a VGG network as its backbone, uses an encoder-decoder in the full convolution layer, and upsamples the feature maps using a bilinear interpolation algorithm in the decoding operation.
(2) DeepLab V3+ improved the Xception. The network combines the ASPP network architecture to extract feature maps at different scales in the image. This captures the fine boundary features of the target and considers global background information. Such a method is beneficial for image segmentation in complex backgrounds. We consider this network for experimental analysis in medical image segmentation for malignant tumor lesions.
(3) U-Net is a proposed method specifically for medical image segmentation, and the network architecture is excellent for scalability and generality. Many designs and improvements (UNet++ [24], UNet 3+ [25]) based on this network have emerged for its application in complex tasks in medical images.

The performance of various classical segmentation methods based on evaluation metrics is shown in Table 3. Among all methods, FCN-8s had the lowest values for each metric. DSC, DeepLab V3+, U-Net, and UNet++ perform comparably, but all are 4 points lower than our method, while UNet3+ is in the second position. From this table, it is evident that our method outperforms all other methods in all six metrics, reflecting its superior performance. It indicates that the supervision of the BKPS module makes the edge attention network achieve the most accurate understanding of both the uncertainty and ambiguity of the tumor boundary.

**Figure 7:**
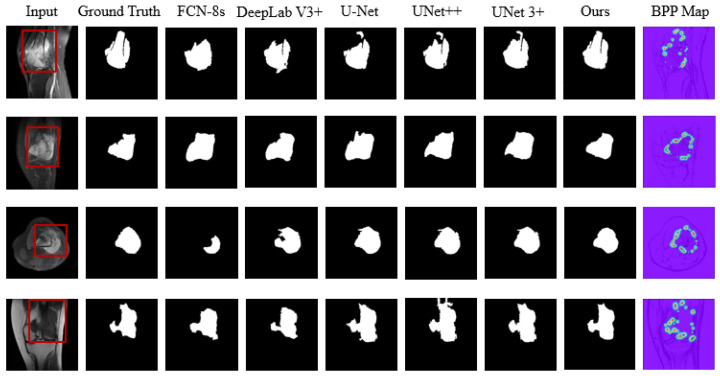
Segmentation results of different classical methods for typical osteosarcoma MRI images.

Figure 7 shows more experimental results. The segmentation effect corresponds to the data in Table 3. Intuitively, our method achieves robust performance in terms of preserving critical edge details and resisting noise interference. The closer the segmentation mask is to the ground truth, the closer the predicted tumor lesion boundary point proposal (BPP) location is to the true tumor border. Further, this confirms the key role played by mixed attention in capturing the contextual dependence of ambiguous boundaries of bone tumor lesions. Through this approach, the model can better understand the ambiguity of the boundary feature representation.

Classical ResNet is used as the backbone in the process of extracting feature maps. In this experiment specifically, we consider the complex relationships between the tumor lesion region and the neighboring tissue, background noise information, and fuzzy boundaries. Therefore, we chose three relatively complex network architectures: ResNet-34, ResNet-50, and ResNet-101. Then, we incorporate the FPN architecture to concatenate the feature maps of the four stages of ResNets and generate feature maps that fuse high-level semantic and low-level feature information.

**Figure 8:**
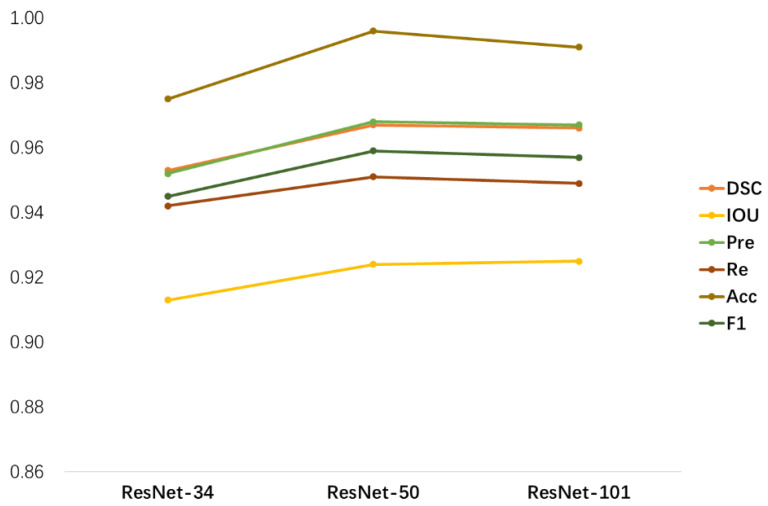
The influence of the three backbones on the overall segmentation performance of SEAGNET according to different metrics.

As shown in Figure 8, the three backbones influence the overall segmentation performance of SEAGNET according to a variety of metrics. ResNet-50 is most adept at segmenting MRI images of osteosarcoma and achieves the best results in this paper. In each of the six metrics, it can be seen that ResNet-50 shows a significant improvement in segmentation performance compared to ResNet-34. There is a slight shift in performance when using ResNet-101 due to the large network scale, which increases training costs, and too many parameters can cause overfitting. Therefore, we adopted ResNet-50 as the backbone based on the considerations of model performance and complexity.

**Figure 9:**
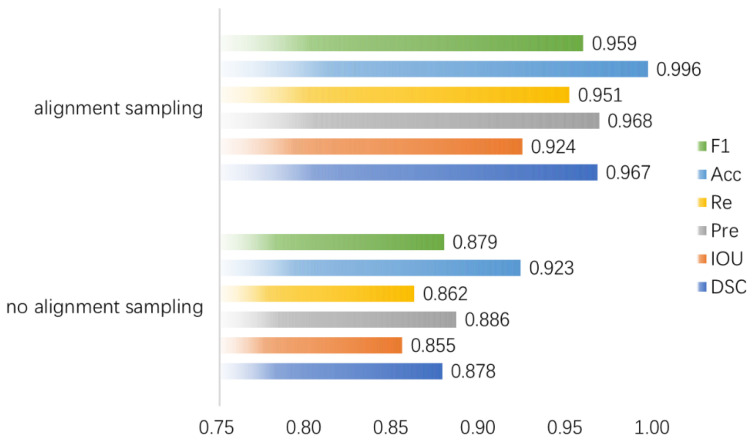
The performance of the overall model for each evaluation metric with and without alignment sampling in the RoI Align operation.

It is necessary to evaluate the effects of alignment sampling on the performance of model segmentation since it is the most essential step in the basic task of feature extraction. In Figure 9 the performance of alignment sampling for each metric is improved by 10% over that without alignment sampling, at offsets of 0.089 for DSC, 0.069 for IOU, 0.082 for Pre, 0.089 for Re, 0.073 for Acc, and 0.080 for F1. The results show that the alignment operation preserves the accurate spatial location information of pixels in the medical image. This is particularly helpful in identifying real tumor boundaries in medical images, enabling the network to capture robust edge detail features, thus leading to more accurate segmentation results.

**Figure 10:**
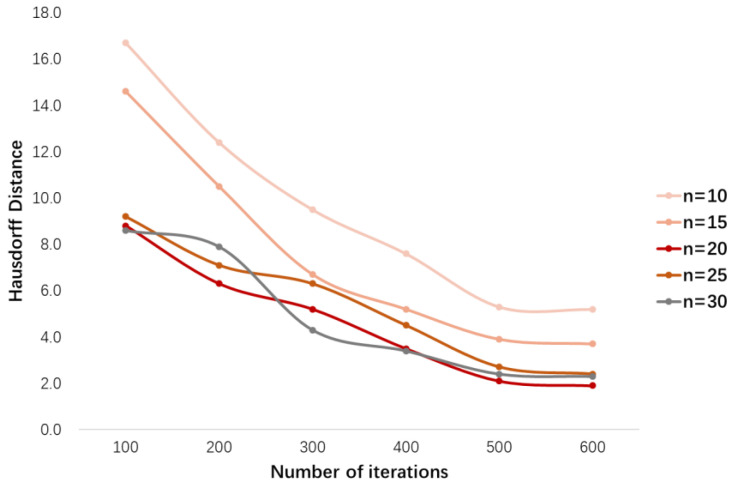
The number of boundary key points *n* takes different values, and the corresponding HD value decreases curve plots.

To select the optimal boundary key points, when training the BKPS module, we randomly select n points on the edges detected by the conventional algorithm (Canny) for the iterative training of Algorithm 1. In our experiments we select $n=\left\{10,\;15,\;20,\;25,\;30\right\}$ for the boundary points optimization. The optimal boundary points $P_{select}$ are selected to generate the output feature maps of the BKPS module, which supervises the learning of the edge attention network. The results in Figure 10 show the change in HD values; the number of iterations is between 100 and 600. When n=20, as the number of iterations increases, the HD value decreases smoothly with a minimum value of 2–3 mm. Because of the ambiguity and uncertainty of the tumor boundary, when n=10 or 15, the segmentation mask more similar to the ground truth cannot be constructed. and when n>20, the decreasing curve of HD value is around that of n=20. Especially for n=30, the falling curve of HD value fluctuates around n=20. The reason is that the number of points is too large, which increases the time complexity of algorithm training. In addition, this will cause more candidate boundary points to gather near the irregular boundary, which will cut off the narrow connection region when constructing the segmentation mask, and the tumor lesion region that exists will be lost. The smaller the HD is, the more similar the segmentation map constructed from the boundary points is to the ground truth. The n points with the smallest HD will be selected as the final result for $P_{select}$.

### 4.5. Ablation Study

We perform ablation experiments to assess the role of each module as a component of the model in the segmentation task. In Table 4 we choose DSC and IOU as the important evaluation metrics. The RoI Align sampling operation preserves the exact spatial position of real key pixels on the tumor edges, bringing an improvement of 4 points, rather than directly pooling to obtain small feature maps leading to the loss of important edge information (DSC is only 0.874).

Next, a mixed attention module (channel attention + spatial attention) is introduced to learn context-rich dependencies, select the important features of bone malignant tumors, and identify boundary contexts in medical images to suppress background noise information, contributing 2 points to the DSC score of the parallel approach. As a comparison, we performed two different serial approaches of channel attention and spatial attention branches, but the DSC score only improved by about 1 point, which proves the superiority of the parallel approach taken in this paper.

The reason for this is that in the “spatial + channel” approach, the spatial attention branch first weighs and extracts features, and then uses the output results as input to the channel attention branch. In this sequential relationship, an overconfident decision about spatial attention occurs. This causes the later attention channel to lose the neighboring correlation of the original features in the spatial dimension, leading to a decrease in segmentation accuracy. Similarly, in the “channel + spatial” serial approach, features based on the channel attention branch are selected first, which filters out important spatial reference information from the channel features and reduces their context-dependent comprehension.

In contrast, the parallel approach explores the rich contextual dependencies of the two dimensions on the basis of the original features separately, preserving the original semantic relationship information in each dimension as much as possible, so as to fully understand the uncertainty and ambiguity of the boundary, and thus obtain the best segmentation performance.

**Figure 11:**
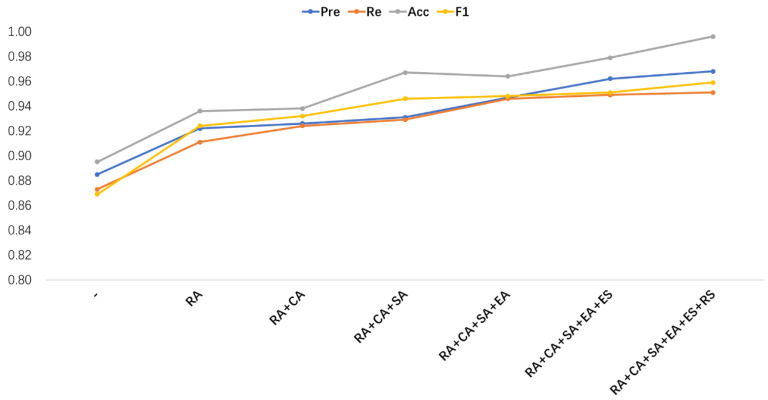
The performance of four metrics, Pre, Re, Acc, and F1 in the ablation experiment. The “-” means the model using no significant components, and the “+” indicates the model with multiple components integrated until the complete SEAGNET model is reached on the far right.

We now focus on edge attention (EA) which enables the network to focus enough on the position information of key pixel points on the edges of tumor lesions, contributing nearly 1 point to the DSC score. However, the fuzzy boundary features of the real lesion regions of osteosarcoma cannot be fully captured yet, or they are missing or redundant. Therefore, the BKPS module supervises (Edge Supervision + Region Supervision) the learning of the EA network so that it can accurately locate the real region boundary of the tumor lesion. The improvement for DSC is 0.02 and for IOU is 0.01, resulting in 0.967 DSC and 0.924 IOU. The data for the remaining four metrics (Pre, Re, Acc, F1) are shown in Figure 11.

For the four metrics, Pre, Re, Acc, and F1, our ablation experimental results also show that the performance of SEAGNET increases smoothly with the addition of each important component. It is worth noting that in the mixed attention module, we use the parallel approach to achieve a significant performance. In Figure 11, we find a significant change in the performance of two metrics: Pre and Acc. RA raises by about 0.04 for all four metrics, indicating the important role of alignment sampling in segmenting the model. The supervision of the BKPS module improves the model by 0.02 for Pre. For Acc, the addition of the mixed attention module improves the model performance by 0.03, and similarly, the supervision of the BKPS module also increases the value of Acc by 0.03. In summary, the contribution of the BKPS module supervision in all six metrics can add 0.01–0.03 to the EA value. The upward trend of these two metrics indicates the effectiveness of our proposed method in segmenting bone malignant tumors with fuzzy boundaries.

**Figure 12:**
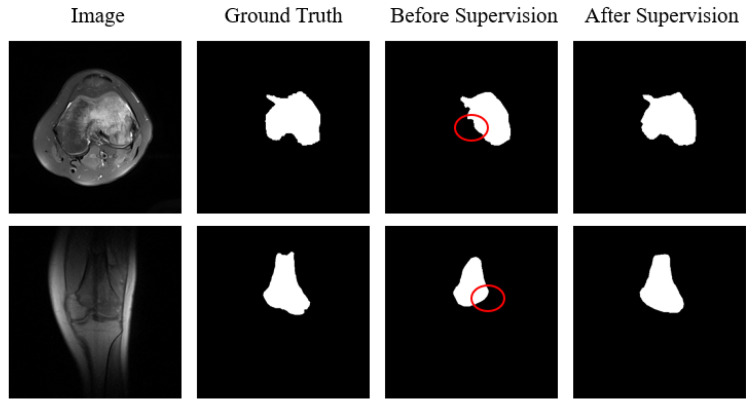
A typical segmentation example compares the segmentation effect before and after applying the BKPS module to supervise edge attention learning. The red circles mark the regions of tumor lesions with ambiguous boundaries that were not captured in the segmentation network before supervision.

Figure 12 illustrates the role of the BKPS module by using a typical segmentation example to demonstrate its effectiveness. Before the BKPS module supervises the segmentation network learning, the segmentation results do not cover the region with ambiguous boundaries, but the region is actually within the tumor lesion. In contrast, the segmentation results are better after supervised learning, which both includes regions with blurred boundaries and portrays as much edge detail as possible, making the segmentation mask closer to the ground truth. This is where the BKPS module comes into play, by supervising edge attention to derive the pixel locations of regions with blurred boundaries.

**Figure 13:**
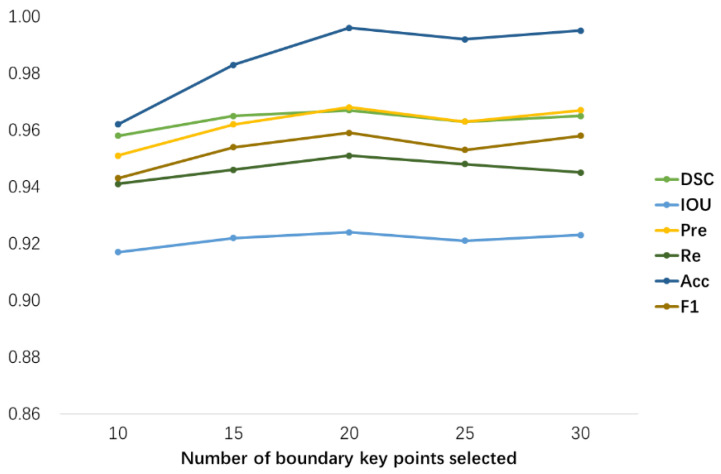
The specific performance of the different number of boundary key points on segmentation evaluation metrics.

In this paper, we perform a quantitative analysis of the iterative training of the BKPS module. We evaluate the impact of the value of the number of boundary key points selection n on the segmentation performance of SEAGNET, as shown in Figure 13. When n=10, medical image segmentation with complex backgrounds loses some details of blurred edges due to insufficient boundary points. This will result in the constructed region not covering the actual tumor lesion area. When the value of n increases to 20, the segmentation performance reaches its best. The values of all six evaluation metrics increased, with Acc being the most significant, improving by approximately 0.03 based on n=10. When n>20, the levels of several metrics show shaking, which is most obvious for DSC, Acc, Pre, and F1. This phenomenon indicates that the increase in the number of key points selected does not bring stability to segmentation performance. In addition, it leads to an increase in training time and computing costs. Therefore, we take n=20 as the optimum in this paper, and the number of iterations of the optimal boundary key point selection algorithm is T=3000. In practice, we adopt a strategy similar to the early stop approach to select the best n value when the HD value between the region constructed by the selected key points and the ground truth is no longer decreasing.

## 5. Discussion

We propose SEAGNET, which has shown excellent results for the segmentation of bone malignant tumor lesions in MRI with complex backgrounds and blurred boundaries. This is attributed to the efficient capture of context-dependent information about ambiguous boundaries through the mixed attention module. This enables the edge attention network to better understand the uncertainty and ambiguity of the boundary features and thus accurately locate the true lesion region and its boundaries. We propose the BKPS module to effectively supervise and correct for the inability of edge attention to accurately segment tumor lesions with complex background relationships.

Most of the existing research methods for MRI images of osteosarcoma remain in traditional image processing methods or machine learning. Although there are some segmentation methods using ResNet or Transformer, these models are too complex and the parameters are too large to train for fitting. The characteristic uncertainty representation of the fuzzy boundary cannot be effectively modeled. The advantage of our approach also lies in the ability to adaptively adjust the complexity of the model according to the difficulty of the target task. Our method can achieve the segmentation of eight images per second with continuous input of MRI images of bone malignant tumors. With good enough hardware equipment, it can process about 20 images in less than 2 s. By the time the doctor views the first image, our method has already completed the segmentation of all the MRI images containing the lesion. This is sufficient for real-time requirements in clinical scenarios. It greatly compensates for shortcomings due to the lack of doctor experience and medical resources. The performance of our proposed segmentation method is comparable to that of a trained doctor.

The limitation of this study is that there is no training and validation of datasets from multiple institutions, as well as of other primary bone malignancy types (Ewing’s sarcoma). This is the main focus of our work in the next stage, and we will further explore making the model applicable to medical image segmentation tasks for other malignant diseases or cancers in the future.

## 6. Conclusions

In this paper, we propose a novel intelligent auxiliary framework network (SEAGNET) for the accurate segmentation of bone malignant tumor lesions. For supervision learning of edge attention, we design a BKPS module that effectively guides the network to identify and localize real locations near the fuzzy boundaries. To preserve the precise spatial location information of key pixels, alignment sampling is performed using the RoI Align operation. The use of mixed attention allows edge attention to be better able to understand the uncertainty and ambiguity of the boundary feature space by capturing rich fuzzy boundary context dependencies. We have extensively validated our model using a real-world medical image dataset. The experimental results show that our method has the best performance levels based on the metrics of DSC (0.967), Pre (0.968), and Acc (0.996). We present an intelligent auxiliary framework that not only effectively increases diagnostic accuracy, improves clinical workflow, and greatly alleviates the dilemma of medical resource shortage and doctors’ inexperience, but also reduces the dependence on manual diagnosis by medical experts according to their domain knowledge, saves time, and improves the overall efficiency of the medical procedure. In the future, we will further improve the robustness of the model and apply it to other cancer segmentation tasks.

## Figures and Tables

**Table 1:** Description of the network layer setup of our proposed SEAGNET.

Network Layers		Parameters Description
	Input layer	(512, 512, 1)
Backbone (ResNet50)	C1 layer	MaxPooling2D (256, 256, 64)
	C2 layer	2 × Identity Block (128, 128, 256)
	C3 layer	3 × Identity Block (64, 64, 512)
	C4 layer	22 × Identity Block (32, 32, 1024)
	C5 layer	2 × Identity Block (16, 16, 2048)
FPN	P2 layer	Conv2D (128, 128, 256)
	P3 layer	Conv2D (64, 64, 256)
	P4 layer	Conv2D (32, 32, 256)
	P5 layer	Conv2D (16, 16, 256)
	P6 layer	MaxPooling2D (8, 8, 256)
	rpn_feature_map	[P2, P3, P4, P5, P6]
	mask_feature_map	[P2, P3, P4, P5]
RPN	class_logits	Conv2D (16, 512 × 512, 2)
	probs	Conv2D (16, 512 × 512, 2)
	bbox	Conv2D (16, 512 × 512, 4)
FC layer		Size(1024)
Head layer		Conv2D (14, 14, 256)
MA layer		Conv2D (14, 14, 256)
BPP layer		Conv2D (14, 14, 256)
EA layer		Conv2D (14, 14, 256)

**Table 2:** Performance statistics of the proposed SEAGNET method compared with existing osteosarcoma segmentation methods based on several metrics. The blanks indicate that the data was not produced.

Methods	DSC	IOU	Pre	Re	Acc	F1
DNN [44]	0.7302		0.7657	0.8612		
Multi-technology combination [45]	0.8984		0.9596	0.8615	0.9802	
DecoupleSegNet [46]	0.9487	0.9051	0.9529	0.9483		0.9500
Residual Fusion Network [47]	0.929	0.867	0.932	0.926		0.929
CaPaN [48]	0.913		0.936			0.932
ETUNet + denoise + CRF [51]	0.935	0.919	0.964	0.949		0.955
Eformer + DFANet [52]	0.964	**0.928**	0.959	**0.955**	0.995	**0.961**
BA-GCA Net [56]	0.927	0.880	0.938	0.937		0.937
SepUNet + CRF + Prop [49]	0.914	0.883	0.937	0.938		0.937
DUconViT [53]	0.924	0.868	0.934	0.937	**0.996**	
PESNet [55]	0.945	0.898	0.940	0.945	0.995	0.945
OSGABN [50]	0.915	0.853	0.915	0.923		0.919
UATransNet Residual [54]	0.921	0.922	0.962	0.945		0.955
SEAGNET (our method)	**0.967**	0.924	**0.968**	0.951	**0.996**	0.959

**Table 3:** Performance comparison of different classical segmentation methods in terms of evaluation metrics.

Model	DSC	IOU	Pre	Re	Acc	F1
FCN-8s	0.834	0.791	0.803	0.827	0.867	0.871
DeepLab V3+	0.920	0.906	0.904	0.872	0.895	0.910
U-Net	0.924	0.919	0.926	0.885	0.901	0.914
UNet++	0.928	0.914	0.907	0.911	0.947	0.932
UNet 3+	0.946	0.920	0.941	0.938	0.926	0.943
Ours	**0.967**	**0.924**	**0.968**	**0.951**	**0.996**	**0.959**

**Table 4:** The performance evaluation of each network component in the proposed model based on two metrics. The network components include RoI Align sampling operation (RA), channel attention (CA), spatial attention (SA), edge attention (EA), edge supervision (ES), and region supervision (RS), the ✓ represents adding the component to the model for experiments.

Description	RA	CA	SA	EA	ES	RS	DSC	IOU
							0.874	0.832
	✓						0.912	0.875
	✓	✓					0.924	0.893
	✓		✓				0.926	0.891
spatial + channel	✓	✓	✓				0.928	0.894
	✓	✓	✓	✓			0.943	0.901
	✓	✓	✓	✓	✓		0.959	0.897
	✓	✓	✓	✓	✓	✓	0.963	0.922
channel + spatial	✓	✓	✓				0.927	0.895
	✓	✓	✓	✓			0.947	0.912
	✓	✓	✓	✓	✓		0.961	0.919
	✓	✓	✓	✓	✓	✓	0.964	0.921
channel and spatial in parallel	✓	✓	✓				0.931	0.910
	✓	✓	✓	✓			0.949	0.916
	✓	✓	✓	✓	✓		0.962	0.923
	✓	✓	✓	✓	✓	✓	**0.967**	**0.924**

## Data Availability

The data used to support the findings of this study are currently under embargo while the research findings are commercialized. Requests for data, 12 months after publication of this article, will be considered by the corresponding author.
